# Norwegian health personnel’s compliance with new legislation on children of ill parents: an exploratory cross-sectional multicentre study

**DOI:** 10.1186/s12913-022-08268-9

**Published:** 2022-09-19

**Authors:** Kristin Stavnes, Torleif Ruud, Jūratė Šaltytė Benth, Ketil Hanssen-Bauer, Bente M. Weimand, Tytti Solantaus, Marit Hilsen, Bjørg Eva Skogøy, Ellen Katrine Kallander, Elin Kufås, Gro Christensen Peck, Bente Birkeland, Kristine Amlund Hagen

**Affiliations:** 1 Nordland Hospital Trust, 8092 Bodø, Norway; 2 Faculty of Medicine, University of Oslo, Oslo, Norway; 3 The Regional Centre for Eating Disorders (RESSP) at Nordland Hospital, Nordland Hospital Trust, Kløveråsveien 1, 8076 Bodø, Norway; 4 Division of Mental Health Services, Akershus University Hospital, Lørenskog, Norway; 5 Campus Ahus, Institute of Clinical Medicine, University of Oslo, Oslo, Norway; 6 Health Services Research Unit, Akershus University Hospital, Lørenskog, Norway; 7 Centre for Mental Health and Substance Abuse, University of South-Eastern Norway, Drammen, Norway; 8 Department of Public Health and Welfare, Finnish Institute for Health and Welfare, Helsinki, Finland; 9 Regional Centre for Child and Adolescent Mental Health, RBUP Øst og Sør, Postboks 4623 Nydalen, 0405 Oslo, Norway; 10 Nordland Research Institute, Postboks 1490, 8049 Bodø, Norway; 11 Vestre Viken Hospital Trust, Drammen, Norway; 12 Stavanger University Hospital, Stavanger, Norway; 13 Sørlandet Hospital Trust, Kristiansand, Norway; 14 Faculty for Health and Sports Science, Department of Psychosocial Health, University of Agder, Grimstad, Norway; 15 Norwegian Centre for Child Behavioral Development, Postboks 7053 Majorstuen, 0306 Oslo, Norway

**Keywords:** Legislation, Law, The Act, Information, Conversations, Children of ill parents, Parental illness, Mentally ill parents, Parents with substance abuse, Physically ill parents, Somatically ill parents

## Abstract

**Background:**

In 2010 the Norwegian Parliament introduced amendments to the Health Personnel Act requiring all health personnel to inform and offer help to their patients’ children and families. We evaluated whether health personnel adhered to their obligations outlined in the Act and investigated whether family and health services characteristics were associated with the degree of compliance with the legislation. Our study was part of a larger Norwegian multi-site study conducted in five health trusts across Norway, assessing the situation for families living with parental illness.

**Method:**

A cross-sectional study using quantitative data obtained from 518 patients 246 children and 278 health personnel was performed. All informants completed a questionnaire, including an instrument corresponding to the obligations in the legislation. Descriptive analyses, factor analysis and logistic regression analysis were used.

**Results:**

The legislation was only partially implemented in the clinics of the health trusts. Compared to estimates prior to the introduction of the new legislation, the situation had improved somewhat, but much work remains to be done to fulfil the obligations decreed by law. The more time-consuming the obligations were, the less often they were met. The substance abuse and mental health services followed up on their obligations to a greater extent than did the physical health services. Conversely, children of physically ill parents were better informed by their families than were children of parents with mental health and substance abuse disorders. When asked the same questions, reports from health personnel were more positive compared to those of children and patients regarding the legislation’s fulfillment.

**Conclusion:**

Data suggest that there has been a change in the support offered to children of ill parents. Additional work is required, however, for the Health Personnel Act to function as fully intended.

## Background

According to the Norwegian Health Personnel Act, §10A, introduced in 2010, hereinafter referred to as The Act, health personnel in Norway are required to ensure that children of patients (CHIP) are offered support. The Act applies to all health personnel, regardless of position or level and type of services, including physical health, mental health and substance abuse health services [60]. Health personnel are required to ask whether their patients have children under 18 years and have a conversation about their parenting capacity, their children’s wellbeing, and about how the children should be informed about their parent’s illness. Children must be given the opportunity to visit their parents during episodes of care, and, if needed, to receive appropriate, age-adjusted information from the health personnel about their parent’s health situation. Health personnel are obliged to refer children to appropriate municipal or specialist health services if deemed necessary. Finally, the work must be documented. Hospitals are required to have child-responsible personnel, to support and coordinate this work [27, 66, 67]. Similar legislation exists in Sweden [42] and Finland [40, 41].

### Previous research relevant to the Norwegian legislation

The rationale behind The Act is the well-established knowledge that CHIP are at elevated risk for experiencing uncertainty and worry and for developing mental health problems, whether the parent has physical, mental or substance abuse disorders [4, 7, 24, 26, 28, 36, 49, 54, 58, 63, 74, 75]. Systematic reviews underline the importance of preventing mental problems for CHIP by giving them information about their parents’ condition, regardless of the type of illness [16, 20, 22, 44].

Prior to the introduction of The Act, independent studies conducted in physical health, mental health and substance abuse services across Norway concluded that there was a lack of systematic support for patients’ children. Furthermore, the support offered was characterized by random and fragmented efforts [1, 17, 68].

Few resources were allocated to the implementation of The Act. Neither economic recourses, guidelines, education, leader involvement, user information nor questionnaires were sufficiently in place nationally, even if some stakeholders had several of these in place at some locations. While child-responsible personnel were tasked with guiding the work in different health departments, they were appointed only to a certain degree [31, 66, 67].

### Differences in reports between informants and type of health service

Reports regarding conversations and information provided are likely to differ depending on the identity of the respondent. Typically, health personnel report that they have provided more information than patients report having received [23, 30, 45]. Several reviews reported that discrepancies in accounts from children and adults (e.g. about children’s mental problems and parenting) are also found frequently [13, 18, 33, 51]. To gain a more nuanced picture, including both parents and children as informants in health care research is often recommended; however, reports from children, in particular, are lacking [39].

As previously noted, reviews of children’s experiences of parental illness have generally described that CHIP are not sufficiently informed, yet we found no research that compared the three health domains in regard to information given to CHIP. Several studies indicated that CHIP with mentally ill or substance-abusing parents are at a somewhat greater risk for mental health problems than CHIP with physically ill parents [3, 34, 46, 65]. This could imply that children with mentally ill or substance-abusing parents are those most in need of appropriate information.

### Family and service factors associated with information and conversation received

A review of Australian intervention programs for children of mentally ill parents found that only three of 20 programs included children under 8 years old [55] which may reflect that health personnel find it more difficult to inform younger children in an age-appropriate manner [39]. Moreover, larger effects of parental illness on externalizing and internalizing problems were found for younger samples in a meta-analysis of children with chronically ill parents [64].

Studies have indicated that people’s educational levels are positively associated with more active information seeking in medical consultations [70] and for health information in general [11]. However, to the best of our knowledge, no studies have identified an association between patients’ educational levels and the information their children receive about their parents’ health condition. We are not aware of any studies examining whether the severity or duration of a parent’s illness is related to the amount of information and number of conversations health personnel had with and about the patients’ children. In their meta-analysis Sieh et al found larger negative effects for children of parents with the longest illness duration [63], and hence their needs for information could be especially important. Studies that have investigated differences between the three health service domains in regard to information / conversations with and about CHIP, are, to the best of our knowledge, lacking.

### Research questions

1. To what degree did health personnel adhere to the following components of The Act: To having conversations with patients, inviting their partners and children to the hospital, informing their partners and children, and documenting this work? How do the different informant groups evaluate the resulting situation?
2. Are there differences between the reports from the different informant groups (child, parent, and health personnel) and between the three service domains?
3. Are different child, patient, family and service characteristics associated with adherence to The Act? The predictors assessed were the children’s age, parents’ educational level, severity of ill parents’ symptoms (physical and mental health), duration of parents’ illness and type of health service.

## Method

### Design

The study was part of the Norwegian CHIP study, a cross-sectional, multicentre quantitative study carried out in five health trusts across Norway. Combined, these health trusts cover specialist health care services for 34% of the Norwegian population. Each health trust comprises several physical, mental health and substance abuse services which are geographically dispersed within the region. Services were informed prior to the initiation of the study.

### Recruitment and procedure

Families were recruited through the ill parent while he/she received treatment in the specialist health services. Only severe physical illnesses are included in The Act. We chose to recruit patients with cancer or severe neurological illness. Units were randomly selected and recruiters visited them on random days. Health personnel were requested to ask all patients with children ages 0-18 years if a study coordinator could inform them about the study. If patients agreed, they were given verbal and written information. We did not have permission to ask patients their reason for choosing not to participate. The data were collected in 2013-2014.

Eligible patients had to be in regular contact (at least every other week) with the participating child and able to read Norwegian. If the patient had more than one child, one of the children was arbitrarily selected, unless the parent voiced a preference regarding which child to include. If the selected child was under the age of 8 years, he or she was not included as a respondent, but rather the parent and health personnel answered questions on behalf of the child. Health personnel responsible for the patient’s treatment were asked to participate if the patient consented to this.

The data collection was conducted at a time and place preferred by the families, usually at their home in the evening. Data were collected with the use of tablets with internet connections linked to a survey database. Research assistants were present during data collection to answer questions and assist with technical issues. For children who had trouble reading, the interviewers read the questions aloud.

### Participants

**Fig. 1:**
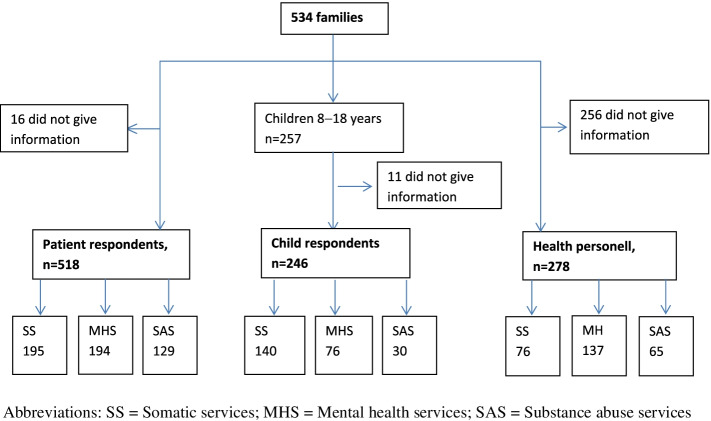
Flowchart of participating respondents. Abbreviations: *SS *somatic services, *MHS* Mental health services, *SAS *Substance abuse services

Participants in this study included 534 families, including 518 patients, 246 children 8-18 years old (hereafter referred to as child respondents) and 278 health personnel. Numbers are unequal because for some families, informants from only one or two of the respondent groups participated. Moreover, 263 children were in the 0-7 age range and, thus, were not administered questionnaires because of their young age. A rather large number of health personnel declined to participate. See Fig. 1 for number of informants and their clinical affiliation.

The mean age of the child respondents was 12.5 years, and for all children ages 0-18 years it was 8.3 years. Of the child respondents 140 (57%) were girls; for children ages 0-18 years, 277 (53%) were girls. Half the children reported becoming aware of their parents’ illness recently / within several months and half reported awareness spanning several years / for as long as they could remember. The patient group comprised 358 mothers (69%) and 160 fathers (31%). Their mean age was 38.1 years. The majority of the patients were ethnic Norwegians (*n*= 483, 93%). Of the patients, 326 (63%) were married or lived together with a partner. For 272 (53%) patients, the other adult in the household was the child’s other biological parent. The patients cared for 2.1 children on average. Moreover, 156 patients (30%) worked full-time, 80 (15%) part-time and 39 (8%) were students, for a total of 275 (53%) patients being active outside home, at least part of the time. Another 127 patients (25%) were temporarily on sick leave. Nearly half the patients (*n*= 240, 46%) received economic support from the Norwegian Labour and Welfare Administration (NAV). The educational level of the patient sample was slightly above the general Norwegian educational level whereas the mean income for the households was slightly below the mean income level for families in Norway (see Table 1 for details). The participating clinicians included 99 nurses (36%), 90 psychologists (32%), 45 physicians (16%), 14 social workers (5%) and 30 others (11%).

**Table 1:** Descriptive statistics of child, patient and family characteristics

Characteristic	Children 8-18 years	Patients (with children 0-18 years)	Health personnel (reporting on children 0-18 years)
Childs age in years, mean (SD)	12.5 (2.9)	8.3 (4.8)	7.9 (4.7)
Education patient, n (%)			
*Elementary school*		102 (19.7)	
*High school*		221 (42.7)	
*College/university*		195 (37.6)	
Income household in NOK, mean (SD)		727,270 (387,368)	
Patient’s physical health score, mean (SD)		45.1 (10.6)	
Patient’s mental symptoms, mean (SD)		20.3 (7.5)	
How long child has known about parent’s illness			
*Got to know recently, n (%)*	30 (12.2)		
*Several months, n (%)*	90 (36.6)		
*Several years, n (%)*	86 (35.0)		
*Have always known, n (%)*	40 (16.3)		
Duration of patient’s illness in years, mean (SD)		8.3 (8.9)	9.2 (8.7)

*Note: NOK* Norwegian croner

### Measures

Data on conversations with and information offered to and received by patients and children were drawn from an instrument developed specifically for the present study. Items were closely linked to the formulations in The Act. This instrument was designed by experienced clinicians and researchers. Table 2 provides an overview of the questions administered to the children, patients and health personnel.

**Table 2:** Frequencies and percentages of legislation components endorsed by children, patients and health personnel

	Children 8-18 years (*n*=246)	Patients (*n*=518)	Health personnel (*n*=278)
***Conversations and documentation about the child: Frequency (percentage) endorsing ‘yes’***		n (%)	n (%)
Was the patient asked whether he/she has children?	N/A	439 (84.7)	259 (94.9)
Did the health care professionals have a conversation with the patient about his/her parental functioning?	N/A	310 (59.8)	237 (86.8)
Did the health care professionals have a conversation with the patient about the child’s situation?	N/A	310 (59.8)	237 (86.8)
Was a questionnaire about the child’s situation used?	N/A	48 (9.3)	95 (34.8)
Was the other adult offered a conversation at the unit?	N/A	272 (52.5)	145 (53.1)
Did the other adult attend a conversation at the unit?	N/A	269 (51.2)	118 (43.2)
Was the child registered in the patient’s electronic record?	N/A	N/A	154 (56.4)
Was a record about the child written?	N/A	N/A	71 (26.0)
Did the discharge letter from the hospital contain information about the child’s needs?	N/A	16 (3.1)	62 (22.7)
***Conversations with the child: Frequency (percentage) endorsing ‘yes’***	n (%)	n (%)	n (%)
Has the patient told the child about his/her illness him/her- self?	228 (92.7)	344 (66.4)	130 (47.6)
Has the other adult told the child about the patient’s illness?	138 (56.1)	N/A	N/A
Did the child visit the unit where the patient was treated?	153 (62.2)	241 (46.5)	94 (34.4)
Was the patient offered a conversation together with the child?	N/A	170 (32.8)	107 (39.2)
Did the child take part in a conversation together with the patient?	69 (28.0)	95 (18.3)	61 (22.3)
Did the health personnel have a conversation with the child alone?	53 (21.5)	49 (9.5)	23 (8.4)
***Evaluation of the resulting situation: Frequency (percentage) endorsing ‘yes’***	n (%)	n (%)	n (%)
Did the child receive sufficient information about the parents’ illness?	187 (76.0)	302 (58.3)	101 (37.0)
Is there openness in the family about the parent’s illness	161 (65.4)	445 (85.9)	184 (67.4)
Are you worried about the child's wellbeing and functioning?	N/A	199 (38.4)	92 (33.7)

*Note*: the other adult is either the child’s other biological parent or the patient’s partner who lives in the household. *N/A* not applicable, the specific question was not administered to that respondent group

Physical health-related quality of life was measured by four items from the SF-8 (Health Survey Short form, REF). SF-8 is a short version of SF-36 and is a widespread and validated instrument [37, 71, 76]. Patients answered the questions based on the last week. For each question, five or six response options were presented. The higher the score, the better the physical health of the patient respondent. Cronbach’s alpha was 0.80 in our study.

Mental health symptoms were measured by the HSCL-10 (Hopkins Symptom Checklist), a short version of the HSCL-90. It comprises 10 questions, four pertaining to anxiousness and six to depressive mood. Patients answered the items based on the previous week, with a four-choice response range. The HSCL-10 is a frequently used and validated questionnaire [25, 69]. The higher the score, the greater the symptomology. In our study, the Cronbach’s alpha was 0.92 for this measure.

### Statistical analysis

Frequencies and percentages were used to investigate the degree of compliance with the legislation, the first aim of the study. The degree of discrepancy/agreement in responses between children, patients and health personnel was also presented by percentages. However, to answer this particular research question we used data only from families for whom corresponding respondents had also given answers and when the same questions were posed. Thus, for these questions the sample sizes are smaller.

To investigate whether child, family and service variables predicted the amount of information and conversation the family was offered, a series of regression models was performed. Because groups of questions typically represent more robust measures than do single items, we performed factor analyses of the questions about conversations and information prior to the regression analysis. This approach leads to considerably fewer regression analyses, and thus fewer tests performed. Questions were categorized by respondent (child, patient, health personnel). Ordinal variables were dichotomized. Factor analysis of correlations among dichotomized questions was performed. *Varimax* rotation was applied, and *oblimin* rotation was tried out, but did not alter the results.

All questions were assessed, but only those factors considered most relevant to our research questions were selected for further analysis. Factor scores were coded as dichotomous variables by assessing the factors’ content. Child, family and service characteristics were entered as predictors into bivariate and multiple logistic regression analyses. The predictors included were children's age, parents' educational level, physical health-related quality of life (SF-8), psychiatric symptom load (HSCL-10), duration of (known) parental illness and type of health service (physical illness, mental illness, substance abuse). Results with p-values below 0.05 were considered statistically significant. Data analyses were performed using SPSS v. 24.

### Missing data analysis

The digital data-collection strategy ensured very little missing data at the individual participant level, as respondents could not move forward in the electronic questionnaires until all items were answered. However, for some of the questions, the response choices included ‘I don't know’ and ‘not applicable’, and these were treated as missing data in factor- and regression analyses. Moreover, responses about income from 90 patients were considered invalid and, therefore, were also treated as missing data.

Some informants were unable to complete the survey. In all, eight child respondents participated without their parent completing the survey, and 11 patients participated without their children ages 8-18 years participating. Only 278 of 518 patients had a participating health personnel respondent and this affected the sample size on which the regression analysis was performed. Missing values in the factor analysis were handled by a logic imputation whenever possible. Informants' factor scores were calculated if the informants had less than 50% missing. Nevertheless, a few factors were included if they were considered to contain decisive answers. For example, if a patient confirmed that a conversation with health personnel about his or her child had taken place, we considered the factor ‘Patients: conversation at hospital’ valid even if the patient had not responded to the question about being asked about his or her children by health personnel. We assumed that the latter must have taken place if the patient endorsed the former.

### Ethical approval

The study was approved by the Regional Committee on Medical and Health Research Ethics South-East (reg.no. 2012/1176) and by the privacy ombudsman at each of the five health trusts taking part in the study. All informants gave written informed consent.

## Results

We first investigated the extent to which health personnel adhered to essential components of The Act. Next, we compared discrepancies in responses given by different respondents and health services. Finally, we examined whether child, family and service characteristics were associated with the degree to which the legislation had taken place.

### Adherence to components of the legislation

The vast majority of patients and health personnel confirmed that the health personnel had asked the patient whether he or she had children; see Table 2. Fewer confirmed that conversations about the patient’s parental functioning and the child’s situation had taken place. In response to a question that only health personnel were asked, even fewer responded that the patient’s child was registered in the patient’s electronic medical record and that a note about the child had been recorded therein. Very few confirmed that the discharge letter from the clinic included information about the child’s needs. Half the patients’ partners were invited to the hospital. Documentation of the work was carried out only to a small extent.

Nearly all child respondents ages 8-18 years, reported that they had been informed of their parent’s illness by their ill parent. For all children, 0-18 years, somewhat more than half of them had been informed. Two-thirds of the child respondents had visited the parent at the hospital, while this was the case for one-third of the children younger than 8 years. Barely a third of the child respondents reported conversations at the hospital together with their ill parent. A fifth of the child respondents had a conversation with health personnel alone. Most of them also had a conversation with their parent present.

### Differences in reports between informants and type of health service

To ensure that this particular question was answered correctly, we reduced the number of respondents to the families where the child, the patient and the health personnel answered. Thus, for these questions the sample sizes are smaller; see Tables 3 and 4.

**Table 3:** ^a^Percentages of legislation component endorsed by patients and health personnel (*n*=273)

	Patients	Health personnel
***Conversations and documentation about the child: Percentage endorsing ‘yes’***	**%**	**%**
Was the patient asked whether he/she has children?	83.5	94.9
Did the health care professionals have a conversation with the patient about his/her parental functioning?	63.0	86.8
Did the health care professionals have a conversation with the patient about the child’s situation?	64.1	84.2
Was a questionnaire about the child’s situation used?	10.6	34.8
Was the other adult^b^ offered a conversation at the unit?	53.5	53.1
Did the other adult attend a conversation at the unit?	52.0	43.2
D**i**d the discharge letter from the hospital contain information about the child’s needs?	2.2	22.7

^a^The sample in this analysis are patients and health personnel for whom both respondent groups are reporting

^b^The other adult is either the child’s other biological parent or the patient’s partner who lives in the household

**Table 4:** ^a^Percentages of legislation components endorsed by children, patients and health personnel (*n* =112)

	Child respondents	Patients	Health personnel
***Information to and conversation with the child, percentage endorsing ‘yes’***	**%**	**%**	**%**
Did the patient tell the child about his/her illness him/her- self?	90	94	71
Did the child visit the unit where the patient was treated?	62	51	42
Did the health personnel have a conversation with the child alone?	22	19	14
Did the health personnel have a conversation with the child and patient?	23	28	30
Did the child receive sufficient information about the parents’ illness?	75	47	30
Is it openness in the family about the parent’s illness	65	78	42

^a^The sample in this analysis are children, patients and health personnel for whom all three respondents are reporting

Children and patients largely agreed on the question of whether the patient had told the child about his/her illness. According to all respondent groups, health personnel’s conversations with the child did not take place in more than one-third of the cases. Patients were more likely than children and health personnel to confirm that there was openness about their illness within the family. Two-thirds of the child respondents reported that they had received sufficient information about their parent’s illness, a higher percentage than reported by patients and health personnel.

In physical health services, most of the patients reported that they had informed their children, while fewer of the patients in mental health services and those in substance abuse services reported the same. The children whose parents were in physical health services reported a higher degree of openness about the illness within the family than did the children whose parents were receiving mental health and substance abuse services. Moreover, according to health personnel, patients in substance abuse services were more likely to have been offered opportunities to have conversations about their parenting and their children’s well-being, and patients in physical health services were the least likely to have been offered such conversations.

### Child, patient, family and health service characteristics associated with adherence to the Act

We included all items related to adherence to The Act, administered to children, patients and health personnel into factor analysis. The questions and the resulting factors were categorized respondent-wise. We selected those factors most relevant to the aims of the study; See Table 5. For the child questions, three factors emerged and we retained the two pertaining to information and conversation: 1) Information/conversation at the clinic and 2) Information/conversation at home. For patients, six factors emerged, of which we kept three factors: 1) Conversations at clinic, 2) Child information at clinic, and 3) Child information at home. For the questions posed to clinicians, seven factors emerged, of which we retained three: 1) Conversation with and information to children, 2) Registration and documentation, and 3) Conversations with patient about parenting and child’s situation. All the questions included in the factors analyses and each factor are displayed in Table 5.

**Table 5:** Factor analyses of items administered to children, patients and health personnel

Factor	Item	**F1**	**F2**	**F3**				
***Children (8-18 years)***								
1 Information/conversation at the clinic	Did you have a conversation together with your ill parent and health personnel?	**0.81**	0.06	0.19				
	Did health personnel treating your parent tell you about your parent’s illness?	**0.81**	0.19	0.04				
	Did you have a conversation with health personnel without your ill parent present?	**0.81**	-0.03	0.01				
	Did you visit the place where your parent receives treatment?	**0.69**	0.01	-0.07				
	*% of total variance explained*	25.0						
	*Cronbach’s alpha*	0.81						
2 Information/conversation at home.	Has another adult with whom you live told you about your parent’s illness?	0.11	**0.71**	-0.12				
	Has your ill parent told you about his/her illness?	-0.10	**0.65**	0.12				
	Are you talking openly about your parent’s illness in the family?	0.27	**0.65**	0.13				
	*% of total variance explained*		15.1					
	*Cronbach’s alpha*		0.46					
3	Do you know of someone to contact if the situation at home becomes difficult?	-0.07	-0.09	**0.75**				
	Is a plan made for what the family can do if the illness gets worse?	0.14	0.15	**0.61**				
	Does your family receive enough help, so you can live normally?	0.05	0.03	**0.47**				
	Do you know enough about your parent’s illness?	-0.07	*0.43*	**0.43**				
	*% of total variance explained*			10.7				
	*Cronbach’s alpha*			0.42				
***Patients***		**F1**	**F2**	**F3**	**F4**	**F5**	**F6**	
1 Conversations at clinic	Did health personnel ask about your child’s situation?	**0.82**	0.14	-0.04	0.06	-0.00	-0.07	
	Did health personnel ask about your parental functioning?	**0.78**	0.12	-0.07	0.10	0.14	-0.02	
	Were you asked whether you have children, on admission?	**0.52**	-0.09	0.07	-0.08	0.09	0.25	
	Were you informed about health personnel’s obligations to contribute to information and support to your child?	**0.51**	0.38	0.02	0.30	0.14	-0.03	
	*% of total variance explained*	22.0						
	*Cronbach’s alpha*	0.69						
2 Child information at clinic	Was a questionnaire about your child’s situation used?	0.14	**0.64**	-0.22	0.30	-0.16	0.07	
	Did your child attend a conversation with you and HP?	0.16	**0.63**	0.26	-0.09	0.21	0.00	
	Did your child visit the unit where you got treatment?	-0.04	**0.58**	0.36	-0.04	0.26	0.05	
	Were you offered a conversation with HP and your child?	0.47	**0.55**	0.09	0.15	0.28	-0.03	
	*% of total variance explained*		7.6					
	*Cronbach’s alpha*		0.66					
3 Child information at home	Has your child received sufficient information about your illness?	0.05	0.11	**0.77**	0.05	0.05	0.17	
	Have you given the child information about your illness?	-0.01	0.30	**0.72**	-0.02	-0.06	-0.24	
	Is it openness about your illness in the family?	-0.04	-0.15	**0.68**	0.08	0.08	0.03	
	*% of total variance explained*			7.1				
	*Cronbach’s alpha*			0.63				
4	Is a plan made for what the family can do if your illness gets worse?	0.09	0.11	0.02	**0.83**	0.00	0.02	
	Is it made clear who the family can contact if your illness gets worse?	0.05	-0.01	0.10	**0.80**	0.17	0.12	
	*% of total variance explained*				5.5			
	*Cronbach’s alpha*				0.67			
5	Did your partner/the other parent participate in a conversation with HP?	0.06	0.14	0.12	0.02	**0.84**	-0.01	
	Was your partner/the other parent offered a conversation?	0.22	0.11	-0.02	0.17	**0.75**	0.01	
	*% of total variance explained*					5.4		
	*Cronbach’s alpha*					0.64		
6	Are you worried about your child’s well-being and functioning? (inversed)	-0.11	0.02	-0.09	0.04	-0.03	**0.86**	
	Has the family received enough help, so that your child’s needs are met?	0.31	0.10	0.20	0.16	0.05	**0.62**	
	*% of total variance explained*						5.2	
	*Cronbach’s alpha*						0.41	
***Health personnel***		**F1**	**F2**	**F3**	**F4**	**F5**	**F6**	**F7**
1	Has the child received sufficient information about the parent’s illness?	**0.81**	0.26	0.07	0.11	-0.09	0.19	-0.04
	Is it openness about the patient’s illness in the family?	**0.77**	-0.10	-0.05	-0.06	-0.03	-0.16	0.03
	Has the child received sufficient information and help?	**0.73**	0.05	0.01	0.01	0.20	0.13	0.36
	Has the patient given the child information about his/her illness?	**0.72**	0.35	-0.19	0.10	-0.16	0.14	-0.08
	*% of total variance explained*	18.1						
	*Cronbach’s alpha*	0.89						
2 Conversation with/information to children	Did the child visit the unit during the patient’s treatment?	0.20	**0.67**	0.05	0.32	0.07	-0.06	0.07
	Did the child participate in a conversation alone with health personnel?	0.08	**0.66**	0.00	-0.24	0.06	-0.13	-0.09
	Did the child participate in a conversation with health personnel and the patient?	0.25	**0.64**	-0.11	0.33	0.18	-0.07	0.22
	Was the patient offered a conversation with you and the child?	0.01	**0.59**	0.26	0.32	0.06	0.28	0.10
	*% of total variance explained*		8.6					
	*Cronbach’s alpha*		0.73					
3 Registration and documentation	Was a questionnaire about the child’s situation used?	-0.10	0.11	**0.78**	-0.08	0.13	0.04	0.23
	Is the child registered in the patient’s electronic record?	0.06	0.01	**0.74**	-0.12	0.05	0.13	-0.17
	Was a note about the patient’s child written in his/her electronic record?	-0.08	-0.03	**0.63**	0.24	0.12	-0.14	0.07
	*% of total variance explained*			7.9				
	*Cronbach’s alpha*			0.65				
4	Was the patient’s partner invited to a conversation with health personnel?	-0.07	0.10	0.07	**0.84**	-0.06	0.15	-0.13
	Did the patient’s partner participate in a conversation with health personnel?	0.13	0.10	-0.10	**0.83**	-0.02	0.08	0.03
	*% of total variance explained*				6.3			
	*Cronbach’s alpha*				0.77			
5 Conversations with patient about parenting and child’s situation	Has the child's situation been discussed with the patient?	-0.2	0.14	0.15	-0.07	**0.88**	0.15	-0.02
	Has the patient's parental function been discussed with the patient?	-0.05	0.05	0.09	0.00	**0.87**	0.11	-0.03
	*% of total variance explained*					5.9		
	*Cronbach’s alpha*					0.81		
6	Is it clarified who the family can contact if the patient’s illness gets worse?	0.08	-0.11	-0.19	0.21	0.16	**0.75**	0.06
	Is a plan made for what the family can do if the patient’s illness gets worse?	0.26	-0.15	0.28	0.06	0.21	**0.67**	0.21
	Did the patient receive information about health personnel’s obligations to contribute to information and support to the child?	-0.08	0.38	0.40	0.05	0.08	**0.61**	-0.14
	*% of total variance explained*						4.5	
	*Cronbach’s alpha*						0.63	
7	Has the family received enough help so that the child’s needs are met?	0.35	0.01	0.16	-0.08	-0.07	0.06	**0.74**
	Are you worried about the child’s well-being and functioning? (inversed)	-0.15	0.11	-0.11	-0.02	-0.01	0.04	**0.69**
	*% of total variance explained*							4.3
	*Cronbach’s alpha*							0.47

Note: Only the factors that were retained and used in the subsequent regression models were given labels

In the child model, children’s age was negatively associated with scores on the factor ‘Information/conversation at the clinic’. That is, older children (8-18 years) reported being less likely to receive information at the clinic where their parents were treated; see Table 6. By contrast, factor scores on ‘Information/conversation at home’ were unrelated to children’s age. Moreover, children who had recently learned about their parent’s illness reported that they received less information at home, compared to children who had always known about it. Children were more likely to be informed at home if their parents reported better physical health and were treated for a physical illness compared to those treated for mental health or substance abuse disorders. However, no significant differences appeared between type of service on the factor scores for ‘Information/conversation at the clinic’. Parents’ educational level did not predict any of the child factor scores.

**Table 6:** Child model (*n* = 246). Results of logistic regression analysis, odds ratio (OR) with 95% CI and *p*-values

Characteristic	Factor 1				Factor 2			
	Information/conversation at the clinic				Information/conversation at home			
	Bivariate models		Multiple model		Bivariate models		Multiple model	
	OR (95% CI)	*p*-value	OR (95% CI)	*p*-value	OR (95% CI)	p-value	OR (95% CI)	*p*-value
Child’s age	0.84 (0.76; 0.93)	**0.001**	0.84 (0.76; 0.94)	**0.001**	1.13 (1.01; 1.28)	**0.039**	1.12 (0.998; 1.27)	0.088
Education patient								
Elementary school	0.51 (0.21; 1.28)	0.152	0.44 (0.16; 1.17)	0.099	0.70 (0.26; 1.88)	0.475	1.60 (0.49; 5.24)	0.442
High school	1.08 (0.60; 1.93)	0.798	0.97 (0.51; 1.86)	0.932	0.81 (0.40; 1.64)	0.557	1.38 (0.59; 3.25)	0.455
College/university	1	-	1	-	1	-	1	-
Income	0.96 (0.90; 1.03)	0.298	0.96 (0.89; 1.04)	0.330	1.11 (1.00; 1.23)	**0.010**	1.10 (0.98; 1.25)	0.106
Patient’s physical health	0.99 (0.98; 1.03)	0.980	0.99 (0.97; 1.03)	0.862	1.02 (0.99; 1.05)	0.331	1.05 (1.01; 1.09)	**0.017**
Patient’s mental symptoms	1.02 (0.98; 1.06)	0.379	1.01 (0.96; 1.05)	0.806	0.95 (0.91; 0.99)	**0.011**	1.02 (0.96; 1.08)	0.559
How long the child has known								
Got to know recently	1.22 (0.43; 3.43)	0.708	0.99 (0.33; 3.02)	0.996	0.15 (0.04; 0.63)	**0.009**	0.20 (0.04; 0.98)	**0.047**
Several months	0.77 (0.34; 1.71)	0.513	0.75 (0.32; 1.72)	0.490	0.50 (0.13; 1.87)	0.303	0.50 (0.12; 2.04)	0.335
Several years	0.87 (0.38; 1.97)	0.731	0.91 (0.38; 2.17)	0.823	0.26 (0.07; 0.97)	**0.044**	0.28 (0.07; 1.12)	0.072
Has always known	1	-	1	-	1	-	1	-
Health care service								
Somatic clinics	1	-	1	-	1	-	1	-
Mental health clinics	1.15 (0.63; 2.10)	0.654	0.90 (0.42; 1.94)	0.785	0.21 (0.10; 0.45)	**<0.001**	0.20 (0.08; 0.54)	**0.001**
Substance abuse clinics	1.03 (0.42; 2.53)	0.948	0.95 (0.33; 2.71)	0.924	0.24 (0.09; 0.66)	**0.006**	0.20 (0.06; 0.68)	**0.010**

*Note*: College/university, has always known, and somatic services are the reference categories. *CI* Confidence interval

According to the patients, the older the child, the more likely he or she had received information at the clinic and at home. This applied for all the children (ages 0-18 years); see Table 7. Patients who reported more severe mental health symptoms, and who were recruited from mental health and substance abuse services were more likely to report having had conversations with health personnel about their children and their own parental functioning. Patients with higher physical health scores and who were being treated in mental health and substance abuse services reported lower scores on the factor ‘Child information at home’. Neither educational level nor duration of illness were significantly associated with any of the factors in the patient model.

**Table 7:** Patient model (*n* = 518). Results of logistic regression analysis, odds ratio (OR) with 95% CI and *p*-values

Characteristic	Factor 1				Factor 2				Factor 3			
	Conversation at clinic				Child information at home				Child information at clinic			
	Bivariate models		Multiple model		Bivariate models		Multiple model		Bivariate models		Multiple model	
	OR (95% CI)	*p*-value	OR (95% CI)	*p*-value	OR (95% CI)	*p*-value	OR (95% CI)	*p*-value	OR (95% CI)	*p*-value	OR (95% CI)	*p*-value
Child’s age	0.99 (0.99; 1.00)	0.226	1.00 (0.99; 1.01)	0.441	1.04 (1.03; 1.05)	**<0.001**	1.04 (1.03; 1.05)	**<0.001**	1.01 (1.00; 1.01)	**<0.001**	1.01 (1.00; 1.01)	**<0.001**
Education patient												
Elementary school	1.35 (0.75; 2.44)	0.319	0.82 (0.42; 1.60)	0.568	0.36 (0.19; 0.65)	**0.001**	0.44 (0.18; 1.08)	0.074	0.98 (0.54; 1.77)	0.945	1.03 (0.54; 1.96)	0.928
High school	1.28 (0.84; 1.96)	0.257	0.90 (0.56; 1.45)	0.663	0.59 (0.37; 0.94)	**0.027**	0.71 (0.35; 1.42)	0.329	0.99 (0.64; 1.51)	0.946	0.97 (0.61; 1.54)	0.886
College/university	1	-	1	-	1	-	1	-	1	-	1	-
Income	0.92 (0.88; 0.97)	**0.002**	0.97 (0.92; 1.03)	0.342	1.10 (1.04; 1.18)	**0.002**	0.99 (0.90; 1.10)	0.990	1.00 (0.95; 1.05)	0.972	1.00 (0.95; 1.06)	0.914
Patient’s physical health	1.01 (0.99; 1.03)	0.133	1.00 (0.98; 1.03)	0.747	0.95 (0.93; 0.97)	**<0.001**	0.96 (0.93; 0.99)	**0.034**	0.99 (0.98; 1.01)	0.516	0.99 (0.98; 1.02)	0.827
Patient’s mental symptoms	1.05 (1.02; 1.08)	**<0.001**	1.04 (1.01; 1.08)	**0.014**	0.98 (0.95; 1.01)	0.174	1.03 (0.98; 1.07)	0.254	1.01 (0.98; 1.04)	0.467	1.01 (0.98; 1.05)	0.440
Duration of illness	1.00 (0.99; 1.00)	0.340	0.98 (0.96; 1.00)	0.114	0.98 (0.96; 0.99)	**0.010**	0.99 (0.97; 1.03)	0.898	0.99 (0.97; 1.01)	0.241	0.98 (0.96; 1.00)	0.090
Health care service												
Somatic clinics	1	-	1	-	1	-	1	-	1	-	1	-
Mental health clinics	2.52 (1.60; 3.96)	**<0.001**	2.09 (1.16; 3.76)	**0.014**	0.13 (0.07; 0.24)	**<0.001**	0.14 (0.06; 0.35)	**<0.001**	1.06 (0.67; 1.65)	0.816	1.40 (0.78; 2.51)	0.262
Substance abuse clinics	4.26 (2.38; 7.62)	**<0.001**	4.73 (2.23;10.03)	**<0.001**	0.10 (0.05; 0.20)	**<0.001**	0.23 (0.09; 0.61)	**0.003**	1.03 (0.61; 1.73)	0.913	1.70 (0.85; 3.40)	0.132

*Note*: College/university, has always known, somatic services are the reference categories. *CI* Confidence interval

According to health personnel, the mental health and substance abuse services were conducting conversations with patients and documenting their work with families to a greater extent than health personnel in physical health services; see Table 8. Health personnel in substance abuse services also reported higher scores on the factor ‘Conversation with and information to children’ than did health personnel in physical health services. Health personnel also reported that conversations with the children occurred more frequently in families with older children and those with more highly educated parents compared to families with younger children and patients with less education. Physical health scores, mental health symptoms and duration of illness were not associated with any of the factors in the health personnel model.

**Table 8:** Health personnel model (*n* = 273). Results of logistic regression analysis, odds ratio (OR) with 95% CI and *p*-values

Characteristic	Factor 1				Factor 2				Factor 3			
	Conversation with and information to children				Registration and documentation				Conversations about parenting and child’s situation			
	Bivariate models		Multiple model		Bivariate models		Multiple model		Bivariate models		Multiple model	
	OR (95% CI)	*p*-value	OR (95% CI)	*p*-value	OR (95% CI)	*p*-value	OR (95% CI)	*p*-value	OR (95% CI)	*p*-value	OR (95% CI)	*p*-value
Child’s age	1.01 (1.00; 1.01)	**0.019**	1.01 (1.00; 1.02)	**0.003**	0.99 (0.99; 0.99)	**0.014**	0.99 (0.99; 1.00)	0.917	0.99 (0.98; 0.99)	**0.009**	1.00 (0.99; 1.01)	0.917
Education patient												
Elementary school	0.36 (0.16; 0.81)	**0.014**	0.21 (0.08; 0.54)	**0.001**	0.89 (0.38; 2.09)	0.782	0.49 (0.18; 1.36)	0.281	1.13 (0.37; 3.47)	0.827	0.45 (0.10; 1.94)	0.281
High school	0.73 (0.39; 1.34)	0.310	0.58 (0.30; 1.15)	0.120	1.42 (0.75; 2.68)	0.282	1.16 (0.57; 2.35)	0.267	0.94 (0.40; 2.19)	0.879	0.55 (0.19; 1.59)	0.267
College/university	1	-	1	-	1	-	1	-	1	-	1	-
Inncome	1.01 (0.94; 1.08)	0.889	0.99 (0.90; 1.08)	0.767	0.91 (0.84; 1.00)	0.054	0.99 (0.89; 1.10)	0.933	0.91 (0.83; 0.99)	**0.046**	0.99 (0.88; 1.12)	0.933
Patient’s physical health	0.99 (0.96; 1.02)	0.387	0.98 (0.95; 1.01)	0.223	1.01 (0.99; 1.04)	0.343	0.99 (0.96; 1.03)	0.394	1.02 (0.99; 1.06)	0.191	0.98 (0.93; 1.03)	0.394
Patient’s mental symptoms	1.01 (0.97; 1.05)	0.621	0.99 (0.94; 1.04)	0.594	1.04 (1.00; 1.08)	0.050	1.02 (0.97; 1.07)	0.675	1.07 (1.01; 1.14)	**0.025**	0.98 (0.91; 1.07)	0.675
Duration of illness	1.01 (0.99; 1.04)	0.344	1.01 (0.98; 1.04)	0.446	1.03 (1.01; 1.05)	**0.037**	1.01 (0.97; 1.05)	0.970	1.07 (1.01; 1.14)	**0.008**	1.00 (0.94; 1.06)	0.970
Health care service												
Somatic clinics	1	-	1	-	1	-	1	-	1	-	1	-
Mental health clinics	1.17 (0.61; 2.24)	0.633	1.88 (0.76; 4.64)	0.171	4.31 (1.78; 10.46)	**0.001**	3.12 (1.07; 9.11)	**0.037**	17.46 (5.67;53.83)	**<0.001**	22.91 (5.21;100.86)	**<0.001**
Substance abuse clinics	1.27 (0.60; 2.71)	0.532	3.34 (1.12; 9.94)	**0.030**	8.00 (3.06; 20.93)	**<0.001**	7.34 (2.12; 25.42)	**0.002**	36.77 (4.75;284.4)	**0.001**	57.12 (5.39;605.11)	**0.001**

*Note*: College/university, has always known, somatic services are the reference categories. *CI* Confidence interval

## Discussion

This study found that health personnel in specialized health care in Norway adhered only to some degree to their obligations regarding giving patients, families and CHIP the conversations and information necessary, according to The Act. The study identified factors that predicted the support given by health personnel.

### Adherence to the components of the legislation

The fact that almost all the patients across all health services were asked whether they had children, indicates a significant shift in health personnel’s practice, as compared to Norwegian reports prior to the legislation [1, 17, 68]. By contrast, only half the children were actually registered in the patient’s medical record, as required by The Act. This lack of documentation suggests workplace barriers [39]. Documentation of this work was for all questions in the study, the aspect that was least followed up. The health care system is completely dependent on documentation, so that important work can be followed up further. A recent Norwegian study, conducted at a psychiatric hospital, reached the same conclusion, as they found that only 56% of patients’ minor children were registered in the patients’ medical records [50]. Moreover, mandated conversations with the patients, were less likely to have taken place, according to our study, and Reedtz et al [50] reported a similar finding, underlining that time-consuming but required work with families was under-prioritized.

Half of the partners were invited to the hospital. The other adult might be the primary childcare provider in this situation, and hence very important to the child. Studies have indicated that partners experience receiving insufficient information about the patient’s illness [21]. Moreover, patients and their partners judge the family's situation differently [35], and thus, partners have their own reactions they need to discuss [6]. We suggest that excluding half of them indicates that a family-focused perspective is lacking.

Most child respondents reported having been informed about their parent’s illness at home. But it could be inferred that the children participating in our study, had to be informed about their parent’s illness, to understand why they might want to participate. Another possibility is that the parents who agreed to participate, were those who had informed their children about their illness. But most of the child respondents told that they had known about the illness for a long period of time. This contradicts that it was this study that contributed to those numbers. A Japanese study reports the same as 72% of the children had been informed about the illness form their ill parent [72].

About one-fifth of the children took part in a conversation at the clinic. Most parents are capable of informing their children themselves. However, there is reason to believe that some health personnel in adult specialized services may not feel fully competent to talk to children about a parent’s illness, and this could be a reason children are not provided with appropriate discussions [53]. Half of the children in our study had lived with their parents’ illness for several years or since they were very young/always, which implies long periods of their childhood; this is the case, especially, for children with parents in substance abuse services. Sieh et al found more significant negative effects for children of parents with the longest illness duration [63]. This should encourage professionals to communicate with their patients’ children, not only through stand-alone conversations, but also with long-term follow-up. Roughly one-third of the child respondents had not received sufficient information and one-third of the parents and health personnel expressed their concerns about the child’s well-being. Hence health personnel did not sufficiently investigate children’s situations in conversations with patients and with their children. Even more children should have been invited to a conversation at the hospital.

Most of the children in our study who had a conversation alone with health personnel also had a talk together with the ill parent. In case the parent is too ill to talk with the child or participate in such a discussion with a professional, we suggest that the other parent or some other trusted person is present for the child to rely on afterwards. The parent should also be told what was said about them to the child once they are better. Dilemmas around contacts between children and their mentally ill and hospitalized parent are discussed in a qualitative paper [43].

### Differences in reports between informants and type of health service

Health personnel reported having more conversations with patients than did the patients themselves. We suggest that the informants may have different understandings of what constitutes a conversation. Clearly, the intention of The Act was to use a prescribed questionnaire [27], which few health personnel used; this might indicate that conversations were less in-depth than patients expected and needed, and could further explain why patients and health personnel reported different numbers of conversations. This supports the findings of earlier studies suggesting that health personnel might overestimate the amount or quality of their interactions with their patients [23, 30, 45].

In general, children reported lower numbers than did their ill parents on information and openness, which indicates that their parents could overestimate their own efforts. Nevertheless, when the informants in the end evaluated the situation, a majority of the children answered that they had received sufficient information about their parent’s illness, contrary to their parents. This is somewhat surprising, given that most of the research on this topic has concluded that CHIP lack information [16, 20, 22, 44].

It is possible that children did not know what information was available, or that they needed some distance from their parent’s problems. Knowing about a parent’s illness does not necessarily mean that the child has enough understanding to cope with the situation. But these reports from CHIP should first and foremost be taken at face value. Children are the best informants about themselves. They are the only ones to know what is enough, i.e. what they can handle. This is exactly the reason why it is important to ask children themselves. A proper level of health literacy for children has not yet been well-established, as several reviews have reported [22, 55, 56], although a whole host of programs are used worldwide today- as described by several reviews from the three health domains [2, 19, 59, 62]. Information, as it is described in the Norwegian Health Personnel Act, is a concept that embraces a wide range of content, form and different levels of emotional support, intended for a wide age span [56]., suggested that current knowledge of children’s needs for information and support seems to be much less developed than general knowledge for parental mental illness. Legislators and clinicians should take this into consideration when demands are placed on all health personnel to inform children.

Almost all children of physically ill parents were informed by their ill parent. This applied to fewer children of parents in mental health and substance abuse services. In the physical health service area, the 10% of the children lacking information were the youngest children, 0-2 years, who cannot be informed, for reasons of maturity and comprehensible language. We suggest that differences regarding stigma for the different patient groups could also exist within families, and might explain the differences in information, which both parents and children corroborate [9, 10, 12, 48, 52, 54]. Children for their part, can develop a sensitivity towards taboo topics [12].

By contrast, the substance abuse services provided the required services to the highest degree, followed by the mental health services. This could be due to a stronger inclination among health personnel in mental health and substance abuse services to include such conversations in the regular care schedule. Another reason could be that children of ill parents in substance abuse and psychiatric services might have greater needs than do children of parents in physical health services, as prior research has suggested [29, 46, 64].

### Child, patient, family and health service characteristics associated with adherence to The Act

The youngest children had visited their parent at the hospital the least. A review of children’s visits to adult intensive care units to see critically ill family members found that such visits were helpful for them to cope with this stressful situation [8]. This is in contrast to the view that children should be protected against such visits. A recent qualitative study [32] on children’s visits to intensive care units, reached the same conclusion: The children felt good when visiting, but desired a more compassionate, caring approach by the nurses, i.e. age-appropriate information and care-taking. The authors advocated for improvements in how to approach visiting children in an individual and caring way. Children’s visits to parents with severe mental illness or severe substance abuse, could also be challenging, and sometimes inadvisable, but they could probably be conducted, in many cases, in the frame of a child-friendly staff. Children’s imaginations often supersedes reality and being able to see how one’s parents is being cared for in a clinic may actually reduce their worry and uncertainty [43].

According to patients and health personnel, the youngest children were less well informed than the older ones at the hospital. As children go through important developmental stages, including the attachment process, during their early years [57, 61], it should be considered vital to give them appropriate contact and/or information that aims to optimize their ability to cope when a parent is ill. One review concluded that *‘children hungry for information were often dismissed as too young to understand’* [22]. We suggest that a gap exists between the knowledge about young children’s development, and the tendency to overlook their need for information and visits to their ill parent [15, 22].

According to the child respondents (8-18 years), age was negatively associated with information received at the clinic. That is, younger children reported being better informed than the oldest children. This could be because the oldest children were already well-informed at home. Or perhaps this finding reflects the fact that teenagers expect more realistic and nuanced information. Several studies suggested that older children, particularly adolescent girls, are more affected by their parents’ illness [47], and are also most concerned about caring activities [14]. The more concerned about and involved with a parent’s illness a child or teenager is, the greater his or her need for information is likely to be.

Children reported that they were better informed when they had known about their parents’ illness for a longer period. We suggest that this is fortunate, because studies indicate that chronicity is a moderator for the transmission of risk to children [5, 63]. However, the need and desire for information may also be very important at the onset of one’s parent’s illness.

The level of patients’ education was positively associated with conversations with children at the hospital only according to the health personnel reports. It seems fortunate that children, for the most part, obtain information when they visit parents in the hospitals regardless of their parents’ educational level.

Higher levels of physical symptoms for the patients were positively associated with information given to children at home, according to child informants and patients. This implies that it is easier to talk about physical symptoms in the family, than it is to discuss psychiatric symptoms or substance abuse. We suggest that this pattern is connected to shame issues [9, 10, 12, 48, 52, 54], as well as difficulties understanding mental health symptoms and putting them into words for children [22, 55, 56].

By contrast, higher levels of mental symptoms, according to the patients, are positively associated with conversations with the patients at the hospital. This indicates that the psychiatric services take their responsibility to have conversations with patients more seriously than do the other services. This might be due to the therapeutic culture.

Children of substance-abusing parents had known about the illness for the longest time. However, they were not as well-informed as children of parents with physical illnesses. A Norwegian qualitative study underlined how children of substance-abusing parents struggled to conduct a normal life inside and outside the family [77]. They are living with a long-lasting and difficult situation, often without sufficient understanding and, thereby, that they are at great risk is not surprising [36, 38].

### Strengths and limitations

A strength of the present study is the inclusion of three different informant groups, all with differing perspectives. Including children in research that is relevant to their situation, especially, is in accordance with the UN Convention on the Rights of the Child. Another strength of the study is the multicentre design covering 34% of the Norwegian population treated in specialized health care facilities. Differences between the health trusts, clinics and regions regarding size and demographics have strengthened representativeness for CHIP in Norway. The inclusion of physical health, mental health and substance abuse services has resulted in a broad knowledge base. Lastly, parts of this study are based on an instrument developed specifically for the study, and the instrument’s strength is that it addresses the specific components of The Act.

A limitation of the present study is a possible selection bias resulting in a sample that is not fully representative of children of ill parents in Norway. We do not have information about the reasons that consent to participate in this study was not given, but we assume that a smaller proportion of those surveyed, confirmed participation. Anecdotal reports from the recruiters suggested that the patients with the highest symptom loads and the most complicated lives were the least likely to participate. Although the data are becoming a bit dated, we still consider them interesting, as they remain the only data to broadly evaluate the outcome of the legislation.

## Conclusions

This study investigated whether health personnel in the specialized health services in Norway, adhered to the amendments of the Health Personnel Act, by which health personnel are obligated to have conversations with patients and their children and partners. The study showed that the specialized health services only partly complied with the new legislation. The more time-consuming and challenging the obligations of the legislation were, the less likely they were to be carried out by health personnel. The health personnel working in mental health and substance abuse services followed up on The Act to a greater extent than did those working in the physical health services.

### Implications

Despite limited economic resources and implementation strategies, the legislation regarding CHIP seems to have been a partial success. Yet greater effort is still needed to ensure that all CHIP in Norway are provided with support when they need it. As most CHIP live with their parents’ illness for a long time, municipalities and specialist health services should bear in mind how critical it is to follow up families with a long-term perspective. Both the youngest and oldest children appear to need more attention from the health care system.

We strongly recommend that research in the CHIP field consider both the patients and their children as informants. To include children in matters that involve their lives is central to the Convention of the Rights of the Child [73]. We also point to the importance of broad studies of different health care services, and from different geographical areas in order to build a foundation for national and international policy-making on an overall level.

## Data Availability

Data are stored at Akershus University Hospital. The dataset cannot be shared as researchers are continuing to work with the remaining data, but data are available from the corresponding author upon reasonable request.

## Abbreviations

CHIP: children of ill parents
NAV: The Norwegian Labour and Welfare Administration
HSCL-90: Hopkins Symptom Checklist
SF-8: Health Survey Short Form
UN: United Nations
